# Evaluating the effectiveness of nicotine replacement therapy in critically ill smokers: A meta-analysis of randomized controlled trials

**DOI:** 10.18332/tid/190798

**Published:** 2024-08-06

**Authors:** Ohoud Aljuhani, Khalid Al Sulaiman, Hadeel Alkofide, Mashael AlFaifi, Asma A. Alshehri, Sarah Aljohani, Haifa Algethamy

**Affiliations:** 1 Department of Pharmacy Practice, Faculty of Pharmacy, King Abdulaziz University, Jeddah, Saudi Arabia; 2 Pharmaceutical Care Services, King Abdulaziz Medical City, Riyadh, Saudi Arabia; 3 College of Pharmacy, King Saud bin Abdulaziz University for Health Sciences, Riyadh, Saudi Arabia; 4 King Abdullah International Medical Research Center, Riyadh, Saudi Arabia; 5 Saudi Critical Care Pharmacy Research (SCAPE) Platform, Riyadh, Saudi Arabia; 6 Saudi Society for Multidisciplinary Research Development and Education (SCAPE Society), Riyadh, Saudi Arabia; 7 Department of Clinical Pharmacy, College of Pharmacy, King Saud University, Riyadh, Saudi Arabia; 8 Drug Regulation Research Unit, College of Pharmacy, King Saud University, Riyadh, Saudi Arabia; 9 Pharmaceutical Care Department, King Saud Medical City, Riyadh, Saudi Arabia; 10 Pharmaceutical Care Department, Prince Sultan Military Medical City, Riyadh, Saudi Arabia; 11 Pharmaceutical Care Services, King Fahad Armed Forces Hospital, Jeddah, Saudi Arabia; 12 Department of Anaesthesia and Critical Care, King Abdulaziz University Hospital, King Abdulaziz University, Jeddah, Saudi Arabia

**Keywords:** nicotine replacement therapy, critical care, meta-analysis

## Abstract

**INTRODUCTION:**

The effectiveness of nicotine replacement therapy (NRT) in critically ill patients remains uncertain, as conflicting research results have been reported. Despite potential side effects and inconsistent data on safety and efficacy, NRT is still prescribed in intensive care units (ICUs) to prevent withdrawal symptoms and manage agitation in patients who are smokers. This meta-analysis aimed to assess the effectiveness of nicotine replacement therapy in critically ill smoking patients.

**METHODS:**

A systematic review and meta-analysis of randomized controlled trials investigated the outcomes of smokers admitted to ICUs and were randomized either to receive or not receive nicotine replacement therapy (NRT) during their ICU stay. The MEDLINE and Embase databases were searched from inception through 13 February 2023 using OVID. The primary outcome was ICU length of stay (LOS) for this systematic review and meta-analysis. Meta-analysis was conducted using both random-effects and fixed-effect models; the latter is recommended when meta-analysis is restricted to just a few studies. The study was registered in the Prospective International Register of Systematic Reviews (PROSPERO) under reference number CRD42023407804.

**RESULTS:**

Of 28 studies initially identified, three, with 67 patients on NRT and 72 controls, were deemed eligible for pooled analysis. Patients who received NRT experienced a shorter LOS (mean difference, MD= -3.06; 95% CI: -5.88 – -0.25, p=0.0, I^2^=0%). The mechanical ventilation (MV) duration was also shorter in the NRT group, but this difference was not statistically significant (MD= -1.24; 95% CI: -3.21–0.72, p=0.22, I^2^=12.69%). Delirium duration was reported by two studies, from which pooled analysis revealed an MD of -0.50 (95% CI: -1.63–0.62, I^2^=0%). The vasopressor duration was assessed in two studies, and the overall MD for vasopressor duration was not statistically different between NRT patients and controls in the fixed-effects model (MD=0.11; 95% CI: -0.75–0.96, I^2^=0%).

**CONCLUSIONS:**

Critically ill smoker patients who received NRT experienced a significantly shorter ICU LOS but no significant differences in the durations of MV, vasopressor use, or delirium.

## INTRODUCTION

Tobacco consumption is a significant cause of preventable deaths worldwide. Many active smokers admitted to intensive care units (ICUs) experience withdrawal symptoms like irritability, anger, anxiety, and depression, as well as behavioral symptoms like restlessness and sleep disturbance^1,2^. Furthermore, these individuals may display increased agitation, try to remove medical devices themselves, require physical restraints, and require higher doses of sedatives, neuroleptics, and analgesics^2^. Withdrawal symptoms caused by neuroadaptation typically begin to manifest a few hours after the last cigarette is smoked and peak within several days to a week^2^.

While nicotine replacement therapy (NRT) has been shown to be effective at alleviating withdrawal symptoms in individuals who quit smoking, its effectiveness and safety in critically ill patients remain uncertain. The physiological effects of nicotine are intricate. Acutely, nicotine leads to elevations in heart rate, blood pressure, and cardiac output, blood pressure, and cardiac output. In the brain, nicotine exhibits both vasodilator and vasoconstrictor properties. Though animal studies suggest that chronic nicotine exposure primarily inhibits cerebral endothelial responsiveness to nitric oxide, which is a potent vasodilator.

The effectiveness of NRT in critically ill patients remains undetermined, as conflicting research results have been reported^7-9^. Despite its potential side effects and inconsistent data on safety and efficacy, NRT is still prescribed in ICUs to prevent withdrawal symptoms and manage agitation in patients who are active smokers^10^.

Several observational studies have investigated the use of NRT in critically ill patients. One study, conducted by Lee et al.^10^, found that ICU patients who received NRT had a higher hospital mortality rate and fewer ICU-free days. On the other hand, another study detected no significant difference in hospital mortality rates between its NRT and non-NRT groups, suggesting that NRT use in the ICU might not cause any apparent harm^8^.

The aim of the current meta-analysis was to determine whether NRT reduces ICU length of stay (LOS) and the durations of mechanical ventilation (MV), vasopressor use, and delirium in critically ill smokers.

## METHODS

The systematic review and meta-analysis were conducted following the 2020 Preferred Reporting Items for Systematic Reviews and Meta-analyses (PRISMA) guidelines^11^. The study was registered in the Prospective International Register of Systematic Reviews (PROSPERO) under reference number CRD42023407804.

### Search strategy and study selection

MEDLINE and Embase databases were searched from inception through 13 February 2023 using OVID. We also searched Clinicaltrials.gov to identify any ongoing or past trials that were not found through other databases. Search terms related to ‘smoking’ and ‘intensive care unit’ were combined with search terms related to smoking cessation. A detailed search strategy can be found in the Supplementary file Table 1.

In this meta-analysis and systematic review, we included randomized controlled trials (RCTs) that involved patients who were active smokers and admitted to an Intensive Care Unit (ICU) and were randomized either to receive or not receive nicotine replacement therapy (NRT) during their ICU stay. Studies meeting these criteria were included regardless of the type, dose, or route of administration of the NRT. Exclusion criteria encompassed non-randomized studies, those involving non-smoker ICU patients, and studies on NRT not conducted in an ICU setting. Studies identified during the initial search were imported into Abstrackr, a tool designed for abstract screening references after removing duplicates^12^. Two investigators (OA and HA) screened all eligible titles and abstracts based on predetermined eligibility criteria. A third investigator (KA) was consulted in cases involving inter-reviewer discrepancy. Studies deemed eligible in the first screening phase were retrieved, and the full text was reviewed independently by two investigators (SA and AA). A third investigator (MA) was consulted in cases of inter-reviewer disagreement.

### Study outcomes

For this systematic review and meta-analysis, the PICO question is defined as P: Critically ill smokers admitted to intensive care units (ICUs); I: The use of nicotine replacement therapy (NRT); C: Placebo or control; O: Influence the length of ICU stay, duration of mechanical ventilation (MV), vasopressor use, and duration of delirium? The primary outcome was ICU length of stay (LOS), measured in days. Secondary outcomes were the durations of mechanical ventilation (MV), delirium, and vasopressor use, measured in days. Pooled analysis was conducted if two or more studies reported sufficient data on the same outcome.

### Data extraction and quality assessment

Two investigators (MA and AS) independently extracted relevant data using a standardized data extraction form. A third investigator (KA) assisted when disagreements arose in the data extracted. Information regarding study design, number of patients, inclusion criteria, intervention, and relevant outcomes were extracted from all included studies.

The Cochrane Collaboration’s tool for assessing the risk of bias in RCTs (RoB2) was used to assess the quality of evidence^13^. This tool includes domains on randomization, randomization process, deviation from the intended intervention, missing outcome data, measurement of the outcome, and the selection of reported results. Two authors (OA and HA) independently scored each study. The GRADE approach was employed to evaluate the certainty of evidence about the risk of bias, inconsistency, indirectness, imprecision, and publication bias^14^. Two authors (HA, KA) independently judged the certainty of the evidence, with disagreements resolved by discussion. A detailed explanation of the domains used in the GRADE approach can be found in the supplementary file Table 2.

### Data synthesis

Since all the study outcomes were continuous variables and were reported using the same scale (days), mean differences (MD) were used to summarize effect estimates, with uncertainty expressed using 95% confidence intervals (CIs). For studies that reported only medians and interquartile ranges, data were converted to means and standard deviations (SD) using the method described by Luo et al.^15^ . If SD was not reported, we followed the recommendations of the Cochrane Handbook for Systematic Reviews of Interventions either by calculating the SD from other available data (e.g. p-values or CIs) or by calculating the average SD when limited information was available to impute the missing SDs^13^.

The meta-analysis used random and fixed effect models, one as primary and the other as sensitivity. The studies were pooled with fixed-effects models, and we used the DerSimonian and Laird estimator for a random effect. Using fixed-effects models has been recommended when a few studies are included in a meta-analysis (e.g. 2 to 5)^16^. Statistical heterogeneity was assessed using I^2^, ≥50%, considered indicative of high heterogeneity. All statistical tests were 2-tailed, and the criterion for significance was p<0.05 by using STATA version 17 SE all statistical analyses.

## RESULTS

### Search and study characteristics

**Figure 1:**
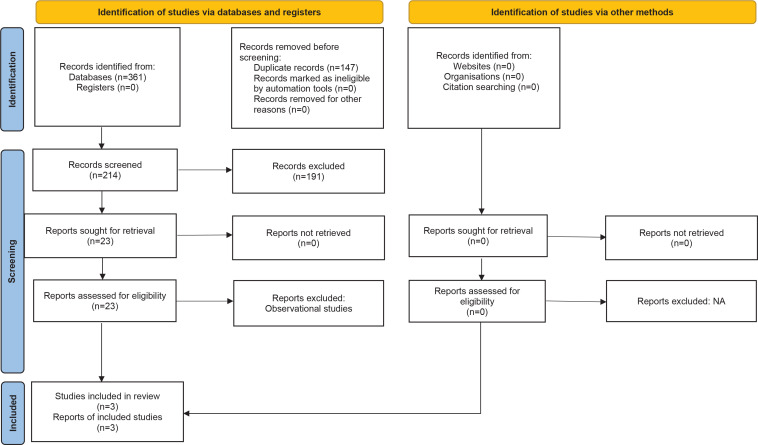
PRISMA flowchart of a systematic review and meta-analysis of randomized controlled trials of smokers admitted to ICUs who received nicotine replacement therapy

A total of 28 studies were identified through our literature search, and after removing 10 duplicates, 18 were initially selected for screening the eligibility. After further screening, just nine studies underwent full-text review, as shown in Figure 1. Three randomized controlled trials (RCTs) were ultimately selected for meta-analysis^17-19^. Collectively, they had 139 patients (67 on NRT, 72 controls), of which 98 were male, representing (70.5%). The characteristics of these three studies are summarized in Table 1. Two studies were double-blinded, with the remaining RCT single-blinded. The outcomes assessed in each study are listed in Supplementary file Table 2. All evaluations focused on comparing patients administered NRT against untreated controls.

**Table 1:** Characteristics of the studies included in the systematic review and meta-analysis of randomized controlled trials of smokers admitted to ICUs who received nicotine replacement therapy (N=3)

*Authors Year Country*	*Number of subjects*	*Blinding*	*Primary outcome*	*Secondary outcomes*
de Jong et al.^17^ 2018 The Netherlands	21 on NRT 26 controls	Double	30-day mortality	Mortality – 90-day ICU and in-hospital ICU and hospital length of stay (LOS) Patient destination at day 30 and 90 (home, ICU, general hospital ward, nursing home, rehabilitation center, deceased) Hours with delirium assessed with CAM-ICU or DOS score Number of nosocomial infections, (serious) adverse events Number of self-removed catheters (e.g. arterial lines, peripheral and central venous catheters, nasogastric tubes, drains, urinary catheters) Number of times patient self-extubates Hours of physical restraints Hours without mechanical ventilation at day 30 [defined as persistent (non)invasive ventilation disconnected for at least 48 h] Cumulative dose of antipsychotics RASS score. Hours with RASS score outside the optimal range (score < -3 or >1).
Pathak et al.^18^ 2013 USA	20 on NRT 20 controls	Double	In-hospital mortality	Delirium Cumulative doses of sedatives and analgesics
Kanova et al.^19^ 2021 Czechia	26 on NRT 26 controls	Single	Incidence of delirium (CAM-ICU)	Duration of sedation, APV, and ICU stay

APV: adaptive pressure ventilation.

### Quality assessment

Using the RoB2 quality assessment tool, one trial^19^ was considered to have a high risk of bias, primarily due to concerns related to the single-blind study design, which raised issues regarding potential deviations from the intended intervention and the measurement of outcomes. On the other hand, the other two studies^17,18^ were considered to have a low risk of bias, as shown in Supplementary file Figure 1.

### Study outcomes

*ICU length of stay*

**Figure 2:**
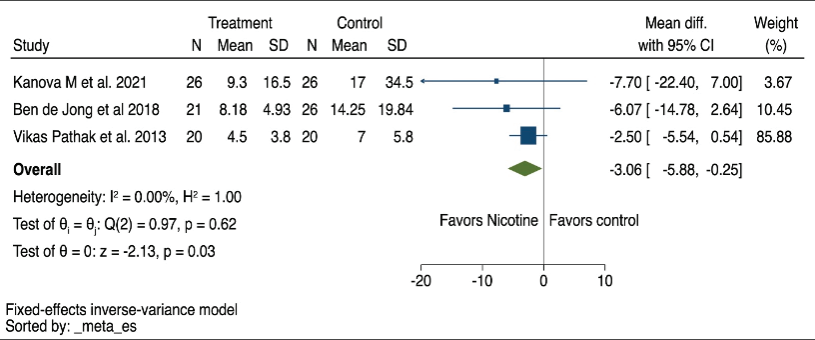
Forest plot of a systematic review and meta-analysis of randomized controlled trials showing the ICU length of stay mean difference using a fixed-effects model in patients receiving nicotine replacement therapy versus controls (N=3)

All three studies in our analysis reported the length of stay in the ICU as their primary outcome. Pooled findings obtained using a fixed-effects model indicate that using NRT reduced ICU LOS, as reflected by a negative overall MD (MD= -3.06; 95% CI: -5.88 – -0.25, p<0.01, I^2^=0%) (Figure 2). Supplementary file Figure 2 shows sensitivity analysis that yielded similar results when a random effects model was employed. The certainty of evidence on this outcome was low due to the serious imprecision and potential for publication bias due to the small number of studies included (Table 2).

**Table 2:** Assessed outcomes of the studies included in the systematic review and meta-analysis of randomized controlled trials of smokers admitted to ICUs who received nicotine replacement therapy (N=3)

*Authors Year*	*Primary outcome ICU LOS*	*Secondary outcome MV duration*	*Secondary outcome Delirium incidence and duration*	*Vasopressor duration*
de Jong et al.^17^ 2018	Reported	Reported	Reported	Not reported
Pathak et al.^18^ 2013	Reported	Reported	Not reported	Reported
Kanova et al.^19^ 2021	Reported	Reported	Reported	Reported

*Duration of mechanical ventilation (MV)*

**Figure 3:**
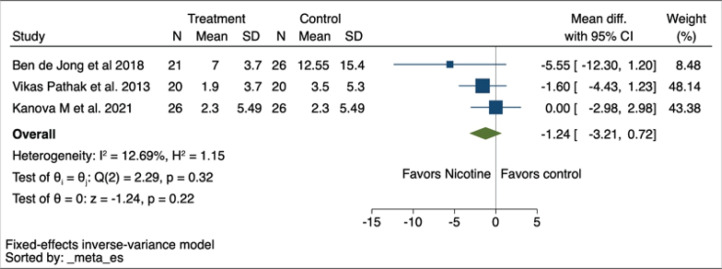
Forest plot of a systematic review and meta-analysis of randomized controlled trials showing MV duration mean difference using a fixed-effects model in patients receiving nicotine replacement therapy versus controls (N=3)

The duration of MV was reported as an outcome in all three studies, with an MD of -1.24 (95% CI: -3.21–0.72, p=0.22, I^2^=12.69%) (Figure 3). These results were consistent with those when a random-effects model was used, as depicted in Supplementary file Figure 3. The certainty of evidence on this outcome was low due to the serious imprecision and potential for publication bias due to the small number of studies included (Table 2).

*Delirium during the ICU stay*

**Figure 4:**
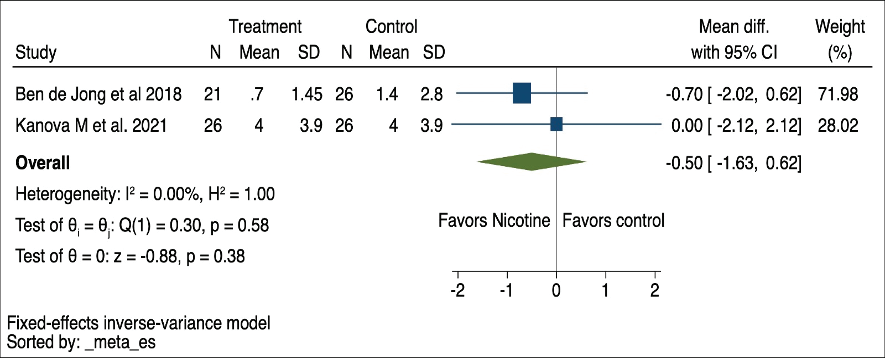
Forest plot of a systematic review and meta-analysis of randomized controlled trials showing the duration of delirium duration mean difference using a fixed-effects model in patients receiving nicotine replacement therapy versus controls (N=2)

Delirium duration was reported by both de Jong et al.^17^ and Kanova et al.^19^, from which pooled analysis revealed an MD of -0.50 (95% CI: -1.63–0.62, I^2^=0%), as depicted in Figure 4. Supplementary file Figure 4 shows that sensitivity analysis using random effects yielded similar results to the fixed-effects model. The certainty of evidence on this outcome was very low due to the serious imprecision and potential for publication bias due to the small number of studies included (Table 2).

*Vasopressor duration*

**Figure 5:**
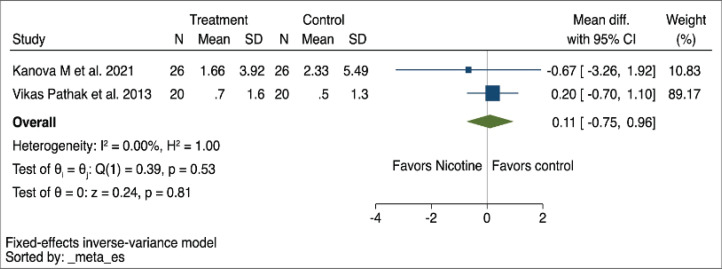
Forest plot of a systematic review and meta-analysis of randomized controlled trials showing the vasopressor duration mean difference using a fixed-effects model in patients receiving nicotine replacement therapy versus controls (N=2)

The vasopressor duration was assessed in two studies (Pathak et al.^18^ and Kanova et al.^19^). The overall MD for vasopressor duration was not statistically different between NRT patients and controls in the fixed-effects model (MD=0.11; 95% CI: -0.75–0.96, I^2^=0%), as shown in Figure 5, and this was consistent in the random effects model as well, as shown in Supplementary file Figure 5. The certainty of evidence on this outcome was very low due to the serious imprecision and potential for publication bias due to the small number of studies included (Table 2).

## DISCUSSION

The current meta-analysis evaluated the effectiveness and safety of nicotine replacement therapy (NRT) in critically ill patients who were active smokers. All findings stem from three RCTs that compared critically ill smokers being treated in ICU administered NRT to reduce their withdrawal from nicotine against counterparts given a placebo. In our meta-analysis, NRT administration was associated with a statistically significant reduction in ICU LOS relative to its non-administration. However, no significant differences were detected in the durations of MV, vasopressor use, or delirium.

Spanning the three studies, using NRT reduced patients’ ICU stay by an average of three days, a finding mostly driven by two studies^17,18^. Contrary to this, in a retrospective study we did not include in our analysis^8^, the NRT group’s length of stay was almost double that of controls (16 vs 9 days, p=0.001). However, in this retrospective study, the NRT group had a higher proportion of individuals with reported severe alcohol abuse, and individuals with an alcohol use disorder not only demonstrated an elevated susceptibility to developing withdrawal syndromes and other medical conditions requiring intensive care, but also were significantly more likely to encounter complications and have prolonged stays in ICUs. Moreover, when it comes to managing agitation and delirium and assessing these conditions, there is a lack of clarity regarding the use of antipsychotic medications, and this may have further confounded these investigators’ findings.

Lee et al.^10^ evaluated the safety of NRT in ICU patients who were active smokers and uncovered no difference between their NRT and control groups in ICU LOS. However, this finding might be due to the study’s small size. Moreover, it is worth highlighting that a significant proportion of ICU admissions involve smokers and that the sudden cessation of nicotine intake can give rise to distressing symptoms, notably agitation and anxiety. Our meta-analysis suggests that integrating NRT into ICU treatment regimens might be relatively effective in reducing ICU length of stay without significant effects on MV and delirium.

Furthermore, it is noteworthy that although the PADIS guidelines provide a comprehensive framework for addressing pain, agitation, delirium, immobility, and sleep disturbances in the ICU, they fail to encompass all potential sources of agitation. Integrating NRT into our practices aligns with the broader objective of minimizing agitation and discomfort, thereby facilitating adherence to PADIS guidelines.

On the other hand, our meta-analysis revealed no significant differences in the durations of MV, delirium, or vasopressor use. That said, these findings mostly derived from a single study^19^. These investigators found that using the Confusion Assessment Method for ICUs (CAM-ICU), there was a significant drop in the average that NRT patients were free of delirium and a reduced requirement for vasopressors. However, the study was small and focused on major surgery patients transferred to the ICU post-operatively. While the use of NRT is considered an appropriate treatment for smokers to mitigate nicotine withdrawal, it is important to acknowledge that its use can lead to certain side effects, including increased risks of delirium, sedation, and prolonged MV, as well as increased risks of cardiovascular, gastrointestinal, respiratory disorders, and insomnia^20,21^. Despite this, data on the safety and efficacy of NRT in critically ill patients remain limited.

Another approach to assessing delirium involves investigating the use of physical restraints and antipsychotic medications. However, only one of the three RCTs included in our meta-analysis examined one of these two outcomes. Gillies et al.^8^ compared the effectiveness of NRT versus no NRT in critically ill smokers and found that delirium and agitation increased significantly with NRT, as assessed using antipsychotic medication (34.1% versus 11.1%, p<0.01) and physical restraints (29.4% vs 9.5%, p<0.01) as proxy outcomes; however, in this study, no validated score was used to quantify delirium^8^. In yet another prospective study, NRT use among smokers in ICUs appeared to contribute to more agitation and delirium^22^. This observational trial focused on physical restraints to evaluate delirium events on a daily basis.

Available cohort studies examining the use of NRT in ICU patients who are presumed to be active smokers have yielded inconsistent findings. Certain studies have identified potentially beneficial effects associated with NRT, while others have uncovered more adverse outcomes, including prolonged periods of MV and even an elevated rate of mortality^18,23^. For instance, in one prospective pilot study, the duration of MV was shorter with NRT in ICU, but the difference was not statistically significant, likely due to the small number of patients in the study^18^. In another study that assessed the safety and efficacy of NRT in critically ill ICU patients with COVID-19, the median number of days free from ventilator usage was zero in both the NRT (IQR: 0–14) and placebo groups (IQR: 0–13)^24^. Notably, this study included ex-smokers who had quit as long as 12 months earlier. Such results are consistent with those of the current meta-analysis, however, statistically significant impact of NRT on MV was detected.

Meanwhile, in a single-center retrospective study, Kerr et al.^25^ found that the duration of MV was significantly prolonged in active ICU smokers who received NRT relative to controls who did not (2.56 vs 1.44 days, p=0.012). Such a discrepancy in findings, relative to our own, could have been influenced by variations in the patients’ baseline characteristics and by the presence of other potentially confounding factors, including active alcohol abuse.

### Strengths and limitations

Our study has notable strengths and limitations. One crucial strength is the strict inclusion criteria we adopted for study selection, including only permitting randomized controlled trials (RCTs), likely resulting in reduced heterogeneity in our findings. Furthermore, we concentrated on a comprehensive set of critical outcomes particularly pertinent to ICU patients. These included the primary endpoint – length of stay (LOS) in the ICU – as well as the secondary endpoints of duration, in days, of delirium, mechanical ventilation, and vasopressor use.

It is nonetheless important to acknowledge the limitations of our meta-analysis. First, we analyzed only a very small number of studies encapsulating very few patients: just 67 on NRT versus 72 controls, which, among other potential effects, reduced both the study’s statistical power to detect inter-group differences and the generalizability of results. Secondly, a certain level of clinical heterogeneity was found among the three studies we examined despite random-effects models being used to produce estimates aligned with fixed-effects models.

## CONCLUSIONS

High-quality data remain sparse and inconclusive on the effectiveness and safety of NRT in ICU patients who are active smokers. Though our meta-analysis revealed a shortening of ICU stays by an average of three days among patients receiving versus not receiving NRT, no significant impacts were detected on the durations of delirium, mechanical ventilation, or vasopressor use. More importantly, the dearth and small size of the published RCTs available to us call both the accuracy and generalizability of our results into question. Larger RCTs remain vital before any empirically supported recommendations can be made on NRT use in ICU patients who smoke.

## Supplementary Material



## Data Availability

The data supporting this research can be found in the Supplementary file.
