# Supplementary material for: Evaluating the effectiveness of nicotine replacement therapy in critically ill smokers: A meta-analysis of randomized controlled trials
Source: Tob Induc Dis. 2024 Aug 6;22:10.18332/tid/190798. doi: 10.18332/tid/190798 (PMC11302334; doi:10.18332/tid/190798)
Supplement: Supplementary file 1 [file TID-22-143-s1.pdf]

Supplementary Materials

Table 1. Search Strategies

Database(s):

- Ovid MEDLINE(R) ALL 1946 to February 13, 2023
- Embase 1974 to February 13, 2023

#	Searches	No. of hits
1	(Smoker* OR Smoking OR Tobacco Smoking OR Tobacco* OR Nicotine OR Tobacco Products OR Smoking Cessation* OR Tobacco Use* OR Cessation* OR Cigar* or Cigarette*).tw.	1068330
2	(Critically ill* OR Critical OR Intensive Care Unit* OR Critical Care OR ICU OR Coronary Care Unit* OR Respiratory Care Unit* OR CCU OR Intensive Care OR Critical Illness OR Multiple Organ Failure).tw.	2575460
3	1 and 2	33383
4	(Nicotine replacement therapy OR NRT OR nicotine replacement OR Nicotine gums OR nicotine patch OR nicotine nasal spray OR nicotine inhaler OR nicotine lozenge OR Tobacco Cessation Product*).tw.	13423
5	3 and 4	361
6	Deduplicate 5	214

Table 2. Explanation for using each domain of the Grading of Recommendations Assessment, Development and Evaluation (GRADE) approach [1]

Domain	Explanation
A – Risk of Bias	Our confidence in an estimate of effect decreases if studies suffer from major limitations that are likely to result in a biased assessment of the intervention effect. For randomized trials, these methodological limitations include failure to generate a random sequence, lack of allocation sequence concealment, lack of blinding (particularly with subjective outcomes that are highly susceptible to biased assessment), a large loss to follow-up or selective reporting of outcomes.<br><br>Every study addressing a particular outcome will differ, to some degree, in the risk of bias. Review authors should make an overall judgement on whether the certainty of evidence for an outcome warrants downgrading on the basis of study limitations. The assessment of study limitations should apply to the studies contributing to the results in the 'Summary of findings' table, rather than to all studies that could potentially be included in the analysis.<br><br>A rating of high certainty evidence can be achieved only when most evidence comes from studies that met the criteria for low risk of bias. The certainty of evidence might be downgraded by one level when most of the evidence comes from individual studies either with a crucial limitation for one item, or with some limitations for multiple items.
B - Inconsistency	When studies yield widely differing estimates of effect (heterogeneity or variability in results), investigators should look for robust explanations for that heterogeneity. For instance, drugs may have larger relative effects in sicker populations or when given in larger doses. If an important modifier exists, with good evidence that important outcomes are different in different subgroups (which would ideally be pre-specified), then a separate 'Summary of findings' table may be considered for a separate population. When heterogeneity exists and affects the interpretation of results, but review authors are unable to identify a plausible explanation with the data available, the certainty of the evidence decreases.
C – Indirect evidence	Two types of indirectness are relevant. First, a review comparing the effectiveness of alternative interventions (say A and B) may find that randomized trials are available, but they have compared A with placebo and B with placebo. Thus, the evidence is restricted to indirect comparisons between A and B.<br><br>Second, a review may find randomized trials that meet eligibility criteria but address a restricted version of the main review question in terms of population, intervention, comparator or outcomes. Other sources of indirectness may arise from interventions studied (e.g. if in all included studies a technical intervention was implemented by expert, highly trained specialists in specialist centres, then evidence on the effects of the intervention outside these centres may be

	indirect), comparators used (e.g. if the comparator groups received an intervention that is less effective than standard treatment in most settings) and outcomes assessed (e.g. indirectness due to surrogate outcomes when data on patient-important outcomes are not available, or when investigators seek data on quality of life but only symptoms are reported). Review authors should make judgements transparent when they believe downgrading is justified, based on differences in anticipated effects in the group of primary interest.
D - Imprecision	When studies include few participants or few events, and thus have wide confidence intervals, review authors can lower their rating of the certainty of the evidence.
E-Publications bias	The certainty of evidence level may be downgraded if investigators fail to report studies on the basis of results (typically those that show no effect: publication bias) or outcomes (typically those that may be harmful or for which no effect was observed: selective outcome non-reporting bias). If a large number of studies included in the review do not contribute to an outcome, or if there is evidence of publication bias, the certainty of the evidence may be downgraded. A prototypical situation that may elicit suspicion of publication bias is when published evidence includes a number of small studies, all of which are industry-funded.

References

1- Higgins JPT, Green S (editors). Cochrane Handbook for Systematic Reviews of Interventions Version 5.1.0 [updated March 2011]. The Cochrane Collaboration, 2011. Available from www.handbook.cochrane.org.

Supplementary Figure 1. Quality assessment results for the three studies included in the meta-analysis, evaluated using the QUADAS-2 evaluation tool

Unique ID	Study ID	Experimental	Comparator	Outcome	Weight	D1	D2	D3	D4	D5	Overall
1	29881956	Y	Y	Y	1	+	+	+	+	+	+
2	23533293	Y	Y	Y	1	+	+	+	+	+	+
3	34506094	Y	Y	Y	1	+	!	+	+	+	-

+ Low risk ! Some concerns - High risk

D1 Randomisation process
D2 Deviations from the intended interventions
D3 Missing outcome data
D4 Measurement of the outcome
D5 Selection of the reported result

Supplementary Figure 2. ICU LOS

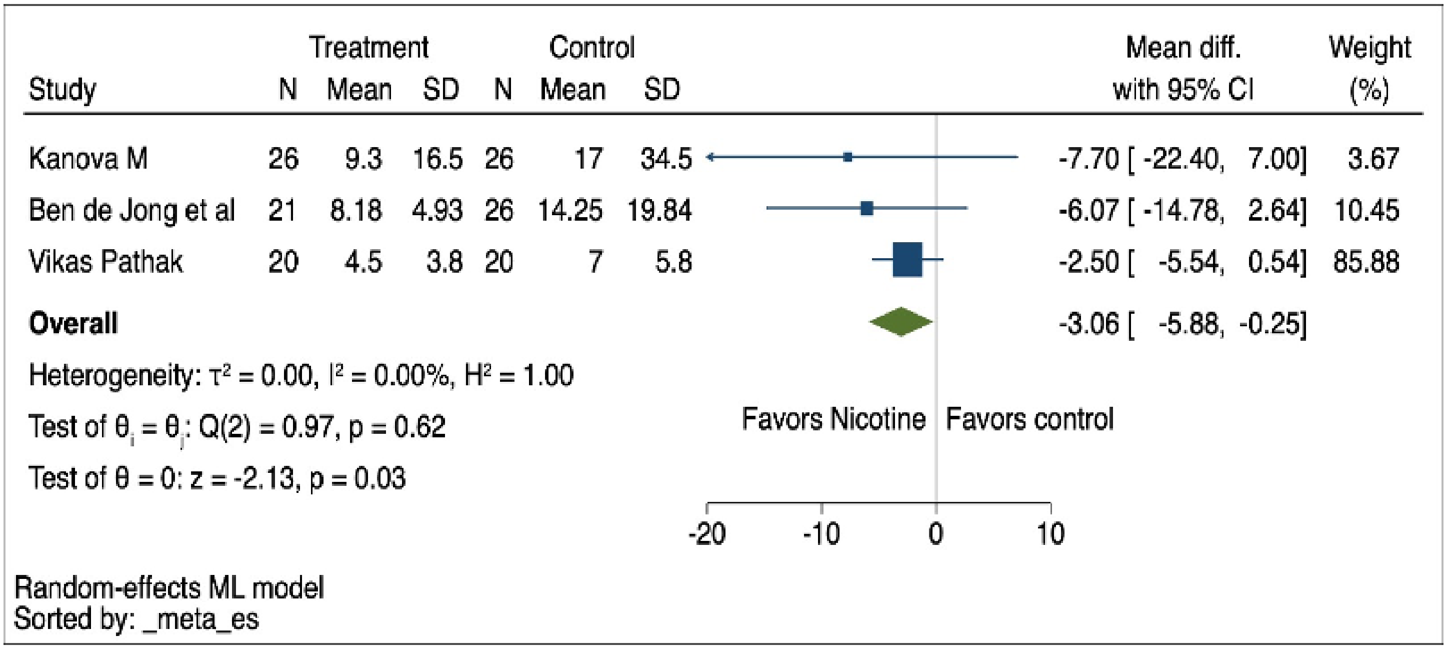

Supplementary Figure 3. MV duration

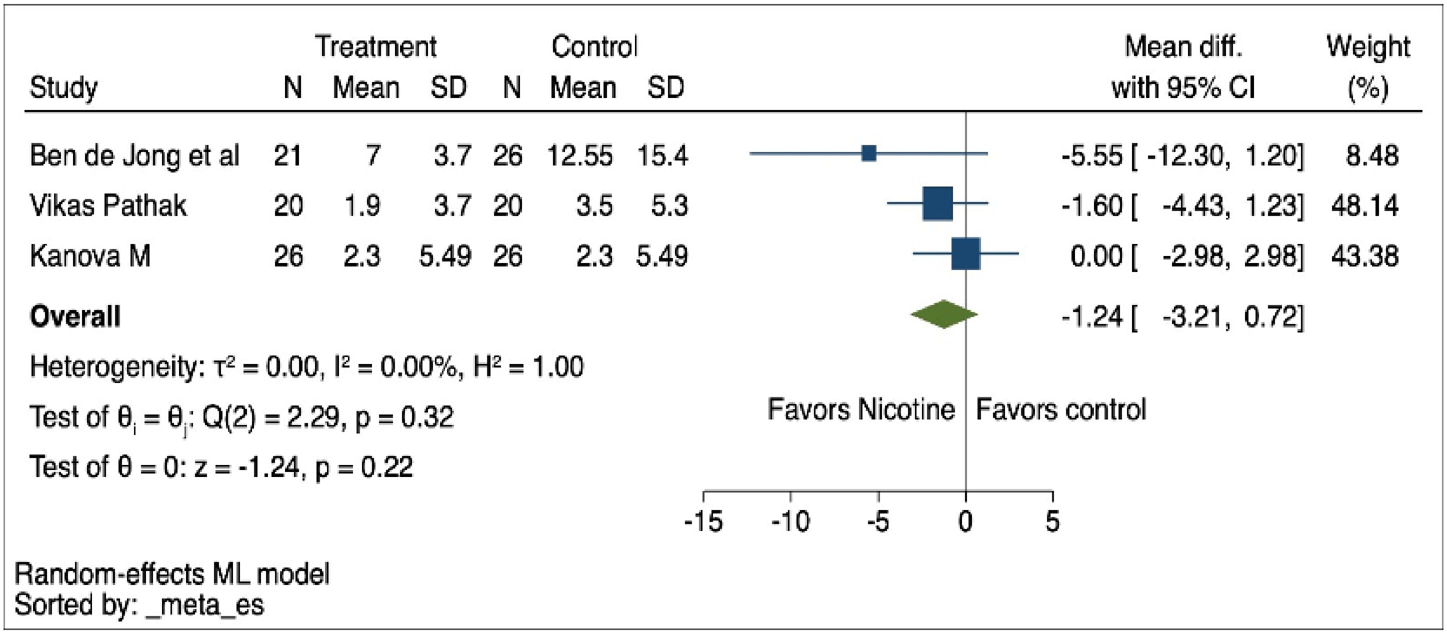

Supplementary Figure 4. Delirium duration

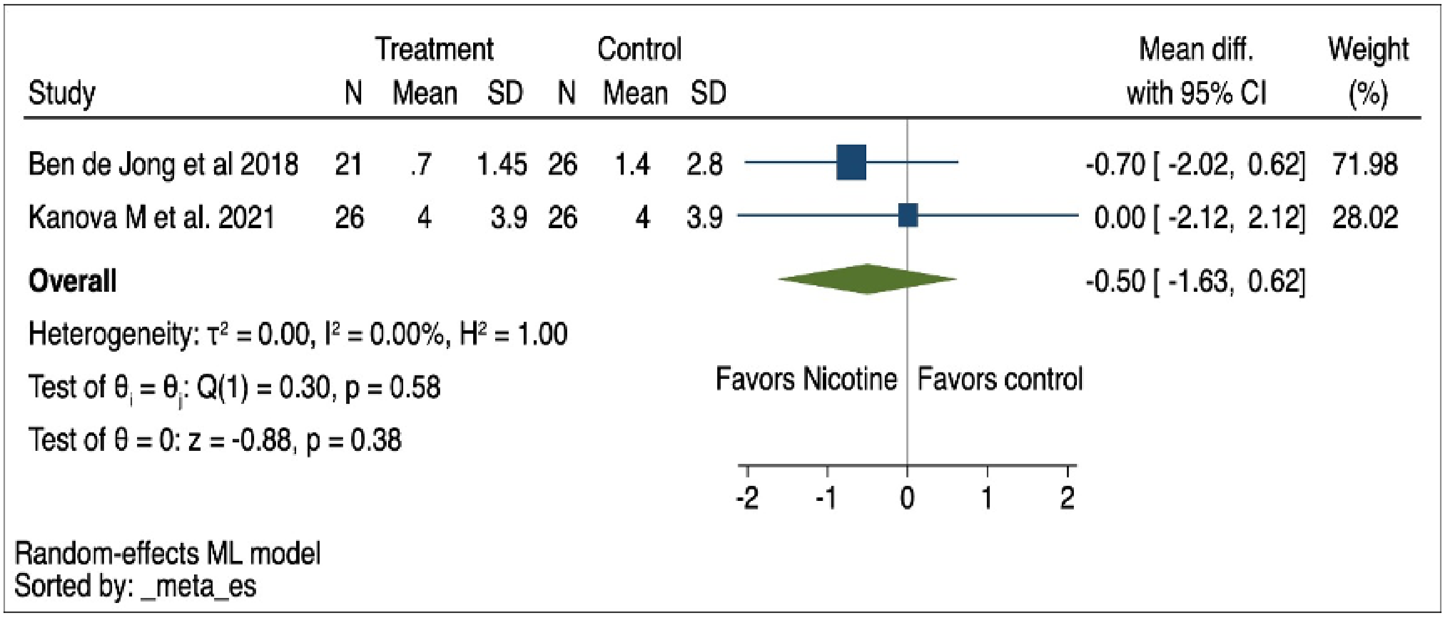

Supplementary Figure 5. Vasopressors duration

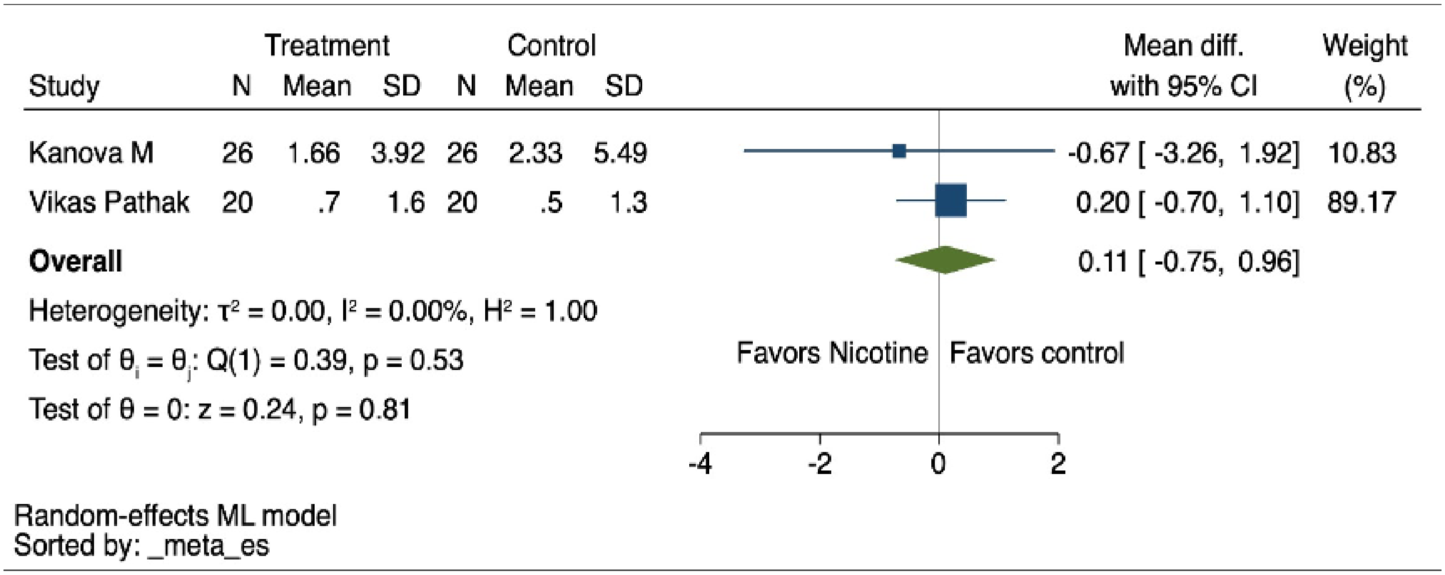

Supplementary Table 2. Summary of findings tables of a systematic review and meta-analysis of Randomized Controlled Trials of smokers admitted to ICUs who received Nicotine Replacement Therapy (N=3).

Outcomes	**Anticipated absolute effects*** (95% CI)		№ of participants (studies)	Certainty of the evidence (GRADE)
	**Risk with Control**	**Risk with Nicotine Replacement Therapy**		
ICU Length of Stay	The mean ICU Length of Stay was **12.75** days	MD **3.06 days lower** (5.88 lower to 0.25 lower)	139 (3 RCTs)	⨁⨁◯◯ Low[a]
Mechanical Ventilation Duration	The mean mechanical Ventilation Duration was **6.12** days	MD **1.24 days lower** (3.21 lower to 0.72 higher)	139 (3 RCTs)	⨁⨁◯◯ Low[a]
Delirium Duration	The mean delirium Duration was **2.7** days	MD **0.5 days lower** (1.63 lower to 0.62 higher)	99 (2 RCTs)	⨁◯◯◯ Very low[b]
Vasopressors Duration	The mean vasopressors Duration was **1.42** days	MD **0.11 days higher** (0.75 lower to 0.96 higher)	92 (2 RCTs)	⨁◯◯◯ Very low[b]

*The risk in the intervention group (and its 95% confidence interval) is based on the assumed risk in the comparison group and the relative effect of the intervention (and its 95% CI).

CI: confidence interval; MD: mean difference

GRADE Working Group grades of evidence

High certainty: we are very confident that the true effect is close to the estimate's effect.

Moderate certainty: we are moderately confident in the effect estimate: the true effect is likely to be close to the estimate of the effect, but there is a possibility that it is substantially different.

Low certainty: our confidence in the effect estimate is limited: the true effect may be substantially different from the estimate of the effect.

Very low certainty: we have very little confidence in the effect estimate: the true effect is likely to be substantially different from the estimate of effect.

Explanations

a. Two points downgraded the certainty of evidence due to serious imprecision, reflected by the relatively wide confidence intervals. In addition, the level of evidence was downgraded due to the small number of studies included in this outcome, which led to a potential suspicion of publication bias.

b. Two points downgraded the certainty of evidence due to very serious imprecision, reflected by the relatively wide confidence intervals. In addition, the level of evidence was downgraded due to the small number of studies included in this outcome, which led to a potential suspicion of publication bias.

©2024 Aljuhani O. et al.
